# The Representation of Suicide on the Internet: Implications for Clinicians

**DOI:** 10.2196/jmir.1979

**Published:** 2012-09-26

**Authors:** Michael Westerlund, Gergo Hadlaczky, Danuta Wasserman

**Affiliations:** ^1^National Centre for Suicide Research and Prevention of Mental Ill-Health (NASP)Department of Learning, Informatics, Management and Ethics (LIME)Karolinska InstitutetStockholmSweden; ^2^Section for Journalism, Media and Communication (JMK)Department of Media Studies (IMS)Stockholm UniversityStockholmSweden

**Keywords:** suicide, internet, websites, pro-suicide, suicide-preventive

## Abstract

**Background:**

Suicide is one of the major causes of death in the world, leading to approximately 1 million deaths per year. While much of what is said about suicide and its causes is still taboo in most contemporary societies and cultures, internet websites and discussion forums have become an important and controversial source of information on the subject. A great deal of ambivalence is discernible as to whether online communication about suicide primarily should be seen as an opportunity or a serious threat.

**Objective:**

To investigate how the subject of suicide is represented on the Internet, based on hits generated by the search engine Google.

**Methods:**

In an exploratory design, Google search results on the target word “suicide”, for the years 2005, 2009, and 2012 respectively, were systematically analyzed and compared.

**Results:**

The study shows that web pages of institutional origin on the subject predominate, that the content provided by these institutions concerns primarily research and prevention, and that the form of communication used by these senders is almost exclusively monological. However, besides these institutional pages there are a substantial number of private senders and pages, often anti-medical and against treatment of depression and other mental problems, characterized by dialogue, confessions and narratives, and to a higher degree, an alternative pro-suicide stance.

**Conclusions:**

To counteract the influence of anti-medical and pro-suicide information, the role of the Internet should be discussed with the patient in clinical practice. Dialogical and confessional communications provide an opportunity for the clinician to gain a deeper perspective into perceptions of patients, regarding both their afflictions and the role of medical treatment in their lives.

## Introduction

Suicide is one of the major causes of death in the world, leading to approximately 1 million deaths per year. It is estimated that by the year 2020, this figure will have increased to 1.5 million [1,2]. While the topic of suicide is still taboo and stigmatized [3], and also often neglected in most contemporary societies and cultures [4], websites and discussion forums have become an important and sometimes controversial source of information on the subject [5,6]. It estimated that there are more than 2 billion Internet users worldwide, with about half of them living in Asia [7]. The application of the Internet in daily life is progressively enhancing. Socioeconomic gaps in Internet use are decreasing, while Internet access is available in more and more rural and remote areas.

One central aspect of the Internet is that the line between private and public communication is expunged. This also has consequences for the development of how the topic of suicide is handled in society. The Internet is not a pretend-world. It is a world populated by real people with different social and cultural backgrounds. In its virtual social environment people meet, exchange thoughts, feelings and experiences; and just like in the real world, the actions people take on the Internet affect their own and/or other people’s lives [8].

A great deal of ambivalence is discernible as to whether online communication about suicide should be seen primarily as an opportunity or a serious threat [9,10]. Some researchers have reflected on the emergence of pro-suicide websites on the Internet [11-14]. It has been pointed out that these sites recommend suicide as a solution to life’s problems and contain detailed descriptions of methods yielding the maximum effect, as well as suicide notes and pictures of people who committed suicide. They persuade and exert group pressure to fulfil suicide plans, glorifying those who have committed suicide. These websites have also given rise to a new form of suicide pact—“net suicides” [15-18]. These developments have given grounds for a fear of the Internet’s detrimental influence on peoples’ understanding and behavior towards suicide. Some authors have put forward that the Internet has a stronger “Werther effect”—ie, a greater potential to induce suicide acts—than other mediated communication forms [19]. Others point to a clear “anti-psychiatric” attitude behind the production of the pro-suicide message [20].

However, the Internet can also be seen as a key resource and a powerful communication tool for understanding and psychologically supporting potentially suicidal individuals [21-24], sometimes referred to as the “Papageno effect” [25]. For example, it may be used to identify people in the risk zone for suicidal acts and then communicate with them, thereby preventing such acts [26].

In April 2005, the word *suicide *generated around 27 million hits on the Google search engine. Four years later, in April 2009, the number of search hits had more than doubled, and in January 2012, the Google search engine generated about 250 million hits on the word *suicide*. The mere existence of so many websites on the suicide subject is not cause for alarm in itself; it means only that the word *suicide *is included in a variety of texts on the Web or that Google’s search algorithms are better at finding texts that include the term *suicide*. Without a more detailed study of these websites’ content, we cannot know what they stand for.

It has been argued that search engines like Google can be seen as meta-sources for information on various topics and that search results on a certain topic may be representative of Internet content at large: “Google search volumes reflect a large proportion of all available Internet search data on the chosen term, thereby capturing a vast amount of information” [27].

The aim of this quantitative study is to examine how the subject of suicide is represented to users on the Internet by proxy of hits for the word *suicide *generated by the search engine Google.

The specific research questions that the study is designed to answer are:

- Who are the senders of the information about suicide on the Internet?
- How is the subject of suicide communicated? Is it primarily through a monological or a dialogical communication?
- What categories of content on the suicide subject appear on the Internet?
- Which discourse about suicide predominates on the Internet?

## Methods

In April 2005, a search was generated on Google (google.com) using the word *suicide. *The first 100 search hits were analyzed systematically. The rationale behind this procedure was to capture a cross-sectional sample of search results. At the heart of Google’s search engine system is the software PageRank [28]. PageRank can be described simply as a type of priority scheme in which more “valuable” and frequently visited websites are ranked higher in the search lists than the less visited and less “valuable” pages. Value is defined by terms similar to that used in the academia, ie, topic relevance and number of citations by other publications (in PageRank this corresponds to links by other websites). The first 100 search results for the word *suicide *should therefore be regarded as the most visited and most cited web pages in the world about suicide. 

The search procedure and analysis were repeated in April 2009 and in January 2012, in order to investigate possible changes in the representation of suicide on the Internet over time.

One aspect that could be argued to influence the results of this study is changes in search engine algorithms used by Google. Such algorithms are optimized by Google frequently to enhance user experience. Changes in these algorithms may affect the search results in a way that does not reflect a change in tangible suicide-related material on the Internet. However, as the aim of this study is to capture what Internet users find on the web, as opposed to describing the complete range of suicide-related materials on the Internet (including those that cannot be found), it is argued that the representativeness of this sample is not threatened: A change in the algorithms affects the results of this study the same way as it affects the results as experienced by users.

After visiting each website, two researchers filled out a checklist with a number of items relating to four pre-specified characteristics, described below. 

Senders: this variable attempted to identify the actor(s) behind the specific text or web page. Authorities associated with a government or a public body were identified, as well as Non-Governmental Organisations (NGOs) and associations, corporations, or private senders.

Form of communication: this refers to the direction of the communication on the given website. Whether users have the opportunity to discuss with each other, comment on/change the contents, or only to receive the contents without interaction. 

Content: this refers to the type of information provided on the website, for example: scientific findings versus specific suicide-related events such as the death of a celebrity, or personal “confessions” and discussions about suicide. 

Discourse: this variable aimed to capture how the topic of suicide was approached. The current medical-psychiatric view, where suicide is seen as harmful behavior, both for the individual and society, and something that is preventable and treatable was contrasted to views promoting/enticing the act of suicide as valid solution to problems in life, and/or suggesting methods and means to commit suicide.

### Exclusion Criteria

Websites in which the word suicide was used metaphorically but that did not otherwise contain any suicide-related content were excluded. Examples of this may be references to members of parliament who have committed “political suicide”, cells in the human body that “commit suicide”. Suicide is a very powerful and symbolically charged word in our culture and is sometimes used in contexts unrelated to the suicidal subject and the act of suicide itself, probably in order to boost the effect of what is expressed [29].

Interestingly, the non-related search hits for the word suicide were especially high for the year 2009 (46%) compared to 2005 and 2012 (13% and 19% respectively) (Table 1). This reflects the volatile nature of Internet-based information content when defined in terms of Google search hits. In this study, the main cause of this increase in non-related search hits in 2009 was firstly an increase in popularity of a pornographic website called *SuicideGirls*, which not even after thorough examination revealed any suicide-related material; and secondly the promotional activities of the electronic punk musical duo called *Suicide *who were releasing a new album as well as starting a tour in 2009. Thus, websites related to both these topics were lacking suicide-related content.

### Attrition

As web pages are removed or modified and because web addresses are constantly changing, the “website attrition” that occurred between period of sampling and analysis was noted in our sample. Time is an important factor affecting attrition in studies of the Internet using a cross-sectional methodology [30, 31]. All websites in this study were visited within 2 weeks of the date of sampling. An attempt was made to analyze even those websites that were unavailable at the time of the visit using Internet Archive (www.archive.org). However, due to the exclusion of non-related hits and website attrition, the sample was thus reduced to 84 hits of full material in 2005, to 48 hits in 2009 and to 82 hits in 2012 (Table 1).

**Table 1:** Included suicide-related content, exclusion due to relevance, and attrition in 2005, 2009 and 2012 respectively. *P*-values based on Fisher’s exact test.

Material	Year			
	2005	2009	2012	*P*-value
	n = 100	n = 100	n = 100	
Included hits	84	48	82	<.01
Exclusion (non-related)	13	46	19	<.01
Exclusion (attrition)	3	6	0	.05

### Coding

Fifty randomly selected search hits were coded by two independent coders in order to test inter-rater reliability. The Cohen’s Kappa value of 0.72 was interpreted as a substantial agreement between the coders [32]. The same coders and measurements were used in the 2005, 2009 and 2012 material.

### Analysis

Considering that this is not a hypothesis-driven study, but rather an attempt at mapping suicide-related content on the Internet, Fisher’s exact was used to establish an association (or the lack thereof) between time and the study variables regarding suicide-related content. Cochran-Armitage trend analysis was used to identify trends in the changes regarding suicide-related content. No significant trends were identified.

## Results

### Senders

The 2005 material shows that 65 (77%) of the 84 Google hits on the search term *suicide *come from *institutional senders *and 19 (23%) from *private senders *(Table 2). The frequencies for 2009 and 2012 confirm that *institutional senders *are the largest category. Yet, the 2005, 2009 and 2012 material has a large proportion of *private senders *in the more relevant search positions (the first ten search hits), which indicates that these senders’ websites are relatively visible and highly attended.

**Table 2:** The share of search hits on Google on the search term suicide distributed on the variable Senders. In percent (and frequencies). *P*-values based on Fisher’s exact test for association (and Cochran-Armitage trend statistic in parentheses).

Senders	Year			
	2005	2009	2012	*P*-value
	n = 84	n = 48	n = 82	
Institutional senders	77% (65)	71% (34)	83% (68)	.27 (.41)
Private senders	23% (19)	29% (14)	17% (14)	

### Form of Communication

The communication about the subject of suicide on the Internet is clearly dominated by the one-way communication form *monologue*, even though the differences between the categories are somewhat smaller in 2009 and 2012 than in 2005 (Table 3).

**Table 3:** The share of search hits on Google on the search term suicide distributed on the variable Form of communication. In percent (and frequencies). *P*-values based on Fisher’s exact test for association (and Cochran-Armitage trend statistic in parentheses).

Form of communication	Year			
	2005	2009	2012	*P*-value
	n = 84	n = 48	n = 82	
Monologue	93% (78)	88% (42)	85% (70)	.30 (.41)
Dialogue	7% (6)	12% (6)	15% (12)	

### Content

*Research and suicide-prevention *is the most common content in the search hits on suicide in all of the studied years. In the 2005 material, 61 (73%) of the search hits fell into this category; in 2009, it was 23 (48%); and in the 2012 material, 61 (74%) of the search hits were categorized as *research and suicide-prevention *(Table 4).

The relatively small share of personal and intimate statements and conversations about suicide, found in the category *confessions*, has been fairly the same for all the three years (Table 4).

**Table 4:** The share of search hits on Google on the search term suicide distributed on the variable Content. In percent and (frequencies). In percent (and frequencies). *P*-values based on Fisher’s exact test for association (and Cochran-Armitage trend statistic in parentheses).

Content	Year			
	2005	2009	2012	*P*-value
	n = 84	n = 48	n = 82	
Research /suicide prevention	73% (61)	48% (23)	74% (61)	<.01 (.82)
Suicide events	2% (2)	6% (3)	9% (7)	.20 (.08)
General reflections	18% (15)	21% (10)	12% (10)	.39 (.33)
Confessions	6% (5)	6% (3)	4% (3)	.78 (.50)
Fiction	1% (1)	19% (9)	1% (1)	<.01 (.97)

### Discourse

The results show that a majority of the search hits for all the studied years have a clear *suicide-preventive *message. In 2005, it was 63 out of 84 hits (75%); in 2009, 27 out of 48 hits (56%); and in 2012, 64 out of 82 hits (78%) (Table 5).

**Table 5:** The share of search hits on Google on the search term suicide distributed on the variable Discourse. In percent and (frequencies). *P*-values based on Fisher’s exact test. *P*-values based on Fisher’s exact test for association (and Cochran-Armitage trend statistic in parentheses).

Discourse	Year			
	2005	2009	2012	*P*-values
	n = 84	n = 48	n = 82	
Suicide preventive	75% (63)	56% (27)	78% (64)	.03 (.20)
Pro-suicide	6% (5)	6% (3)	0	<.01 (.053)
Both categories	11% (9)	21% (10)	9% (7)	.12 (.78)
Non-applicable	8% (7)	17% (8)	13% (11)	.33 (.42)

## Discussion

Although the *institutional senders *quantitatively dominate the communication about suicide on the Internet, the relatively large proportion of non-institutional *private senders *illustrates how the Internet effects the way that suicide is communicated in today’s society.

The analysis of the relation between the variables *senders *and *form of communication *in the 2005, 2009, and 2012 material shows that communication about suicide on the Internet by *institutional senders *is almost entirely in a *monological *form, with very few instances of *dialogue*. In contrast to this, the proportion of the communication form *dialogue *for the *private senders *constitutes 21% in 2005, and in 2009 and 2012, the *dialogue *represents more than a third of the search hits (36%) for these senders. So, today there is a clear distinction between the *institutional senders *and the *private senders *in terms of how to communicate the subject of suicide.

*Dialogue *is thus a quite common form of communication about suicide for the *private senders*. The Internet, uniquely, consists of several different forms of mass communication, where the interactive multi-way communication is the part that means something qualitatively new to the suicide subject, while the one-way monological communication on the Internet stands for an extension of an already existing institutional communication about the suicide subject. The contents of the dialogic communication are based on the social reality outside the Internet. They are shaped and conventionalized according to the specific factors of the communication that occur in the virtual environment, such as a higher degree of anonymity, a greater spatial distance and, to a large extent, a text-based communication.

The most common content on the subject of suicide on the Internet is about *research and suicide-prevention*. The analysis of the relation between *senders *and *content *in the 2005, 2009 and 2012 material shows that communication about suicide from the *institutional senders *is made up mainly from texts on *research and suicide-prevention *(82%, 62%, 82% respectively). This content category also makes up a substantial proportion for the *private senders *(39%, 14%, 36% respectively), but here the categories *confessions *and *general reflections *also make up a noteworthy share (for *confessions *22%, 21%, 21% respectively).

The content category *confessions *contain personal and intimate stories and conversations about one’s own or other people’s suicidal acts and suicidal thoughts. When reading these texts more closely, it is clear that the people writing them are asking questions about the meaning of their existence, that they are crying out for help, that they seek confirmation and understanding, and that they are trying to support and encourage each other the best they can, and in some cases even urge each other to carry out suicidal plans.

According to other studies regarding the representation of suicide on the Internet, pro-suicide websites have shown to be high up on search engines’ result lists, making them highly visible and accessible for those who are looking for suicide-related material [33,34]. These findings are congruent with this study’s results for the years 2005 and 2009, but it is not as clear in the 2012 material. Another recent study also points out that the preventive websites are more visible and accessible on the Internet today compared to those that are pro-suicide [35]. This does not necessarily mean that pro-suicide websites have declined in numbers; they’re just not as prevalent among the first 100 search results, when using the more general search term *suicide*. This could be due to a recent and deliberate strategy (eg, search engine optimization) from suicide-preventive actors to make their websites more reachable and popular, and thereby get a better ranking in the search results lists. Hopefully it is an increased awareness about the risks that pro-suicide websites may have on vulnerable Internet users that lies behind this strategy. These types of websites play a documented role in information on various types of suicide methods, the glorification of individuals who have committed suicide, as well as encouragement to go through with suicide plans [36-40]. Before the development of the Internet, pro-suicide texts were unusual and difficult for audiences to get hold of. The expansion of the Internet has thus enabled pro-suicide messages to spread and can consequently be received by more people today than before. Thus, for an Internet user searching for suicide-related material using a search engine, the likelihood, or risk, of unintentionally finding pro-suicide messages is lower in 2012 than in 2009 and 2005. However, users consciously aiming at finding pro-suicide material will have no problem in doing so [41].

### The Challenge for Clinicians

The results show that it is institutional actors, distributing contents of research and suicide-prevention in a monological way, that dominate the communication about suicide on the Internet. But besides these institutional senders, there is a considerable proportion of private actors for which the Internet has meant the opportunity to publish material and discuss, confess and seek contact on a subject that has always been strongly taboo and therefore “belonged” to only a few voices in public discourse. This opportunity has resulted in both constructive and strongly destructive contributions, such as pro-suicide messages.

Today, it seems a very important task for clinicians to respond to the substantial amount of pro-suicide messages on the Internet and to continue to develop preventive strategies and resources for individuals at risk for suicidal acts [42]. The preventive resources should also be developed using the *dialogic *communication between the mental-health expertise and help-seeking individuals; unidirectional information about mental illness and suicide prevention is not enough [43]. Another important task is to raise awareness among clinicians about the risks it may pose to suicidal individuals to visit pro-suicide websites, which contain descriptions and evaluations of potent suicide methods and promote an attitude that suicide can be an acceptable way to solve life problems. A key instrument could be to establish routines in which clinicians ask their patients about their use of the Internet and also help the patients to find preventive sites with therapeutic resources.

### Conclusion

Through the Internet, the dominant understanding of suicide is challenged by new voices, and the battle over definitions of “right and wrong” and perceptions of “true and false” has intensified. The possibility of reaching out to large groups of users is no longer monopolized by institutional senders. These developments brought on by the wide spread use of the Internet have posed obstacles as well as benefits in the field of suicide prevention.

Clinicians taking part in the online dialogical communication, on platforms where patients/laypersons discuss mental health-related topics, could be a helpful tool in the development of cohesion between patient and clinicians [44], and increase the understanding of the patient perspective. 
